# Role of salicylic acid glucosyltransferase in balancing growth and defence for optimum plant fitness

**DOI:** 10.1111/mpp.12906

**Published:** 2020-01-21

**Authors:** Yudai Kobayashi, Noriho Fukuzawa, Ayaka Hyodo, Hangil Kim, Shota Mashiyama, Tsuyoshi Ogihara, Hirofumi Yoshioka, Hideyuki Matsuura, Chikara Masuta, Takeshi Matsumura, Minoru Takeshita

**Affiliations:** ^1^ Laboratory of Plant Pathology Faculty of Agriculture Department of Agricultural and Environmental Sciences University of Miyazaki Japan; ^2^ Bioproduction Research Institute National Institute of Advanced Industrial Science and Technology (AIST) Sapporo Japan; ^3^ Laboratory of Plant Pathology Graduate School of Agriculture Kyushu University Fukuoka Japan; ^4^ Graduate School of Agriculture Hokkaido University Sapporo Japan; ^5^ Graduate School of Bioagricultural Sciences Nagoya University Nagoya Japan; ^6^ Present address: Ehime Research Institute of Agriculture, Forestry and Fisheries Fruit Tree Research Center Matsuyama Ehime Japan

**Keywords:** benzothiadiazole (BTH), defence, fitness, growth, immune induction, salicylic acid, salicylic acid glucosyltransferase

## Abstract

Salicylic acid (SA), an essential secondary messenger for plant defence responses, plays a role in maintaining a balance (trade‐off) between plant growth and resistance induction, but the detailed mechanism has not been explored. Because the SA mimic benzothiadiazole (BTH) is a more stable inducer of plant defence than SA after exogenous application, we analysed expression profiles of defence genes after BTH treatment to better understand SA‐mediated immune induction. Transcript levels of the *salicylic acid glucosyltransferase* (*SAGT*) gene were significantly lower in BTH‐treated *Nicotiana tabacum* (Nt) plants than in SA‐treated Nt control plants, suggesting that *SAGT* may play an important role in SA‐related host defence responses. Treatment with BTH followed by SA suppressed *SAGT* transcription, indicating that the inhibitory effect of BTH is not reversible. In addition, in BTH‐treated Nt and *Nicotiana benthamiana* (Nb) plants, an early high accumulation of SA and SA 2‐*O*‐β‐d‐glucoside was only transient compared to the control. This observation agreed well with the finding that *SAGT*‐overexpressing (OE) Nb lines contained less SA and jasmonic acid (JA) than in the Nb plants. When inoculated with a virus, the OE Nb plants showed more severe symptoms and accumulated higher levels of virus, while resistance increased in *SAGT*‐silenced (IR) Nb plants. In addition, the IR plants restricted bacterial spread to the inoculated leaves. After the BTH treatment, OE Nb plants were slightly larger than the Nb plants. These results together indicate that *SAGT* has a pivotal role in the balance between plant growth and SA/JA‐mediated defence for optimum plant fitness.

## Graphical abstract

*SAGT*, whose transcript levels can be suppressed by BTH, plays an important role in balancing plant growth and defence for optimum plant fitness.

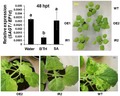

## 1 INTRODUCTION

The trade‐off between growth and defence in plants has been thought to be regulated by signal pathways that are mediated by plant hormones (Huot *et al.*, 2014). The phytohormone salicylic acid (SA) is involved in plant development and responses to biotic and abiotic stresses (Rivas‐San Vicente and Plasencia, 2011). Most SA in cells is glucosylated and/or methylated (Rivas‐San Vicente and Plasencia, 2011). The conversion of methyl salicylate (MeSA) to SA can be catalysed by SA‐binding protein 2 (SABP2) to induce systemic acquired resistance (SAR) (Forouhar *et al.*, 2005; Park *et al.*, 2007; Tripathi *et al.*, 2010), a key mechanism in the innate immune system of plants (Canet *et al.*, 2010). On the contrary, SA can be converted into SA 2‐*O*‐β‐d‐glucoside (SAG) (Pastor *et al.*, 2013). The glucosylation of SA is mediated by uridine diphosphate (UDP)‐glucosyltransferase, also known as SA glucosyltransferase (SAGT) (Vlot *et al.*, 2009), which is generally not required to induce SAR (Loake and Grant, 2007). Seto *et al. *(2011) reported that *SAGT* of tobacco also catalyses the glucosylation of tuberonic acid (12‐hydroxyjasmonic acid; TA), which is a derivative of jasmonic acid (JA), and its expression can be induced by mechanical wounding. *SAGT* expression is induced by exogenous SA application or pathogen attack, indicating a crucial role for SAGT in regulating a balance between SA and SAG. Chivasa and Carr (1998) previously reported that SA‐induced resistance was greatly reduced by the expression of NahG, which converts SA to catechol, in a transgenic *Nicotiana tabacum* line (*NahG* Nt), indicating that the resistance depends on the concentration of endogenous SA. The effect of exogenously applied SA on plant growth is often affected by plant species and growth stage, and varies with frequent changes in endogenous SA levels controlled by SAGT (Vlot *et al.*, 2009; Klessig *et al.*, 2018).

Chemically induced immune responses have been well studied and documented for developing strategies to protect plants from pathogen attack. Although the effectiveness of resistance‐inducing chemicals varies widely depending on the combination of host and pathogen, such immune induction is generally accompanied by up‐regulation of defence genes associated with SAR (Ryals *et al.*, 1996; Oostendorp *et al.*, 2001; Gozzo and Faoro, 2013; Faoro and Gozzo, 2015; Dempsey and Klessig, 2017). For synthetic immune induction, benzothiadiazole (BTH), also known as 1,2,3‐benzothiadiazole‐7‐thiocarboxylic acid‐*S*‐methyl‐ester (ASM), is one of the most commonly used chemical inducers. BTH can induce host resistance against a wide range of pathogens, including plant viruses (Ishii *et al.*, 1999; Narusaka *et al.*, 1999a, 1999b; Anfoka, 2000; Pappu *et al.*, 2000; Oostendorp *et al.*, 2001; Cools and Ishii, 2002; Smith‐Becker *et al.*, 2003; Mandal *et al.*, 2008; Lin and Ishii, 2009; Takeshita *et al.*, 2013; Frąckowiak *et al.*, 2019). This immune induction by BTH is correlated with an increase in gene expression of resistance‐related genes, including the *pathogenesis‐related protein 1* gene (*PR1*), a well‐known marker of SA‐induced SAR. Tripathi *et al. *(2010) reported that SABP2 catalyses conversion of BTH into acibenzolar and that acibenzolar is required for SAR induction in tobacco.

Friedrich *et al. *(1996) showed that BTH increased the transcript level of *PR1a* in a dose‐dependent manner, even in *NahG* Nt, and did not directly induce SA accumulation in the plants. These results indicate that BTH can elicit the resistance‐related pathway downstream of SA accumulation. BTH can induce resistance to *Peronospora tabacina* and tobacco mosaic virus (TMV) in *NahG* Nt, suggesting that it can activate defence responses without SA accumulation (Friedrich *et al.*, 1996). Lawton *et al. *(1996) first reported that BTH can induce host resistance in *NahG Arabidopsis* plants but not in the *Nim1* (*NPR1*; *NON‐EXPRESSOR of PATHOGENESIS‐RELATED GENES 1*) mutant, suggesting that BTH can activate the SAR‐associated pathway between SA accumulation and NPR1 expression. SA has been found to bind NPR1, which has a slightly higher affinity for BTH than for SA (Wu *et al.*, 2012).

These findings raise the question: How can BTH induce the immune response more effectively than SA? We first analysed an inhibitory effect of BTH on viral infection and then analysed the changes in transcript level of the genes associated with host basal resistance. We eventually focused on the changes in transcript level of salicylic acid glucosyltransferase (*SAGT*) as the potential cause of the difference in action between BTH and SA. Here, we found that BTH suppressed *SAGT* expression to enhance immune induction and that *SAGT* can discriminate between SA and BTH to induce resistance. In addition, we propose that *SAGT* regulates not only SA but also JA, two major plant hormones controlling plant defence. We conclude that *SAGT* is a key factor that modulates the balance (trade‐off) between plant growth and defence.

## 2 RESULTS

### 2.1 Prior treatment with BTH suppresses CMV‐inducing symptoms

**Figure 1:**
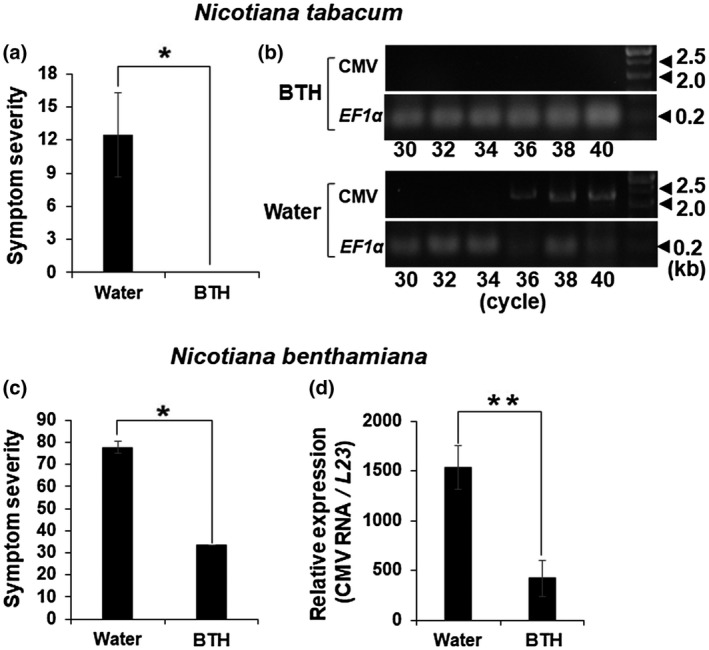
Mean disease severity and relative levels of cucumber mosaic virus strain Y (CMV‐Y) RNA in benzothiadiazole (BTH)‐treated and water‐treated plants. (a) and (c) Mean symptom severity (±*SE*), (b) relative levels of CMV‐Y RNA3, and (d) those of CMV‐Y RNA3 and RNA4 in the wild‐type (Wt) *Nicotiana tabacum* (Nt) and Wt *N. benthamiana* (Nb) plants. BTH (0.12 mM) was applied 2 days before inoculation with CMV. Symptoms on the second and third upper, noninoculated leaf tissues were evaluated and then the leaves were collected at 9 days post‐inoculation (dpi) for Nt and 5 dpi for Nb. Relative accumulation levels of viral RNA were measured (b) by reverse transcription (RT)‐semiquantitative PCR for Nt and (d) by RT‐quantitative PCR for Nb using total RNAs from the leaves of the plants. Severity levels (in arbitrary units) and CMV RNA levels (in arbitrary units) are for individual Nt (*n* = 6) and Nb (*n* = 3) plants. *Significant difference (*p* < .05) and **significant difference (*p* < .01) between the BTH‐treated and the water‐treated controls, according to Student's *t* test, respectively

When wild‐type (Wt) *N. tabacum* (Nt) and *Nicotiana benthamiana* (Nb) plants were treated with BTH 2 days before inoculation with cucumber mosaic virus (CMV), the plants developed very mild symptoms (Figure 1a,c). CMV accumulated to a much lower level in the BTH‐treated Nt plants than in the control at 9 days post‐inoculation (dpi) (Figure 1b), and to less than one‐third the level of the control in BTH‐treated Nb plants at 5 dpi (Figure 1d). These results suggest that BTH is certainly effective in suppressing viral spread to upper leaves. Treatments with BTH did not induce any abnormal development in the Nt and Nb plants.

### 2.2 BTH and SA differ in their mechanism of action in immune induction

**Figure 2:**
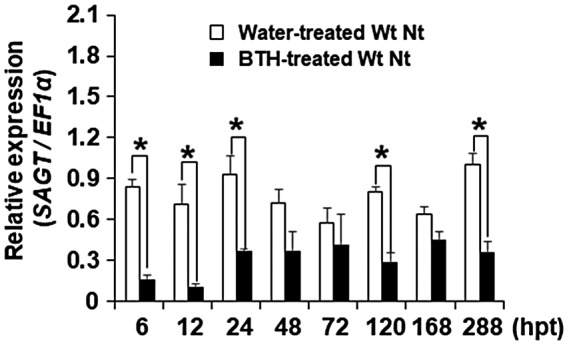
*SAGT* transcript levels in benzothiadiazole (BTH)‐treated wild‐type *Nicotiana tabacum* plants (Wt Nt) as determined by quantitative reverse transcription PCR. Leaf samples were harvested from plants in a greenhouse at 6–288 hr post‐treatment (hpt) with BTH (0.12 mM). Black bar: BTH‐treated, white bar: water‐treated. Levels (in arbitrary units) are for individual plants (*n* = 3). *Significant difference (*p* < .05) between the BTH‐treated and water‐treated leaves according to Student's *t* test. Bars indicate standard errors (±*SE*)

To characterize the chemical induction of immunity by BTH, we analysed the expression of several marker genes involved in SA‐, jasmonic acid (JA)‐, and ethylene (ET)‐signalling, SA accumulation, and RNA silencing in BTH‐treated leaf tissues of Wt Nt and *NahG* Nt plants. The transcript levels of these genes were measured using quantitative reverse transcription PCR (RT‐qPCR) between 6 hr post‐treatment (hpt) and 288 hpt. Accumulation of *PR1a* and *PR1b* transcripts in BTH‐treated plants was significantly higher than in water‐treated plants at 12 hpt (Figure S1a,b), but other host genes (*SAGT*, *Coi1*, *PDF 1.2*, *EREBP1*, *EREBP2*, *ERF1*, *PAL*, and *ICS*) were not up‐regulated by the BTH treatment compared with those in the water‐treated controls (Figures 2 and S1c–i). Furthermore, the relative levels of the *RDR1* and *RDR6* transcripts were not consistently elevated by the BTH treatment (Figure S1j,k). Obvious differences in gene expression were not found between the *NahG* Nt and the Wt Nt plants (Figure [Link], [Link], [Link]a–l). These results suggest that the host responses activated by BTH in the SA‐mediated signal transduction pathway are regulated downstream of SA biosynthesis.

**Figure 3:**
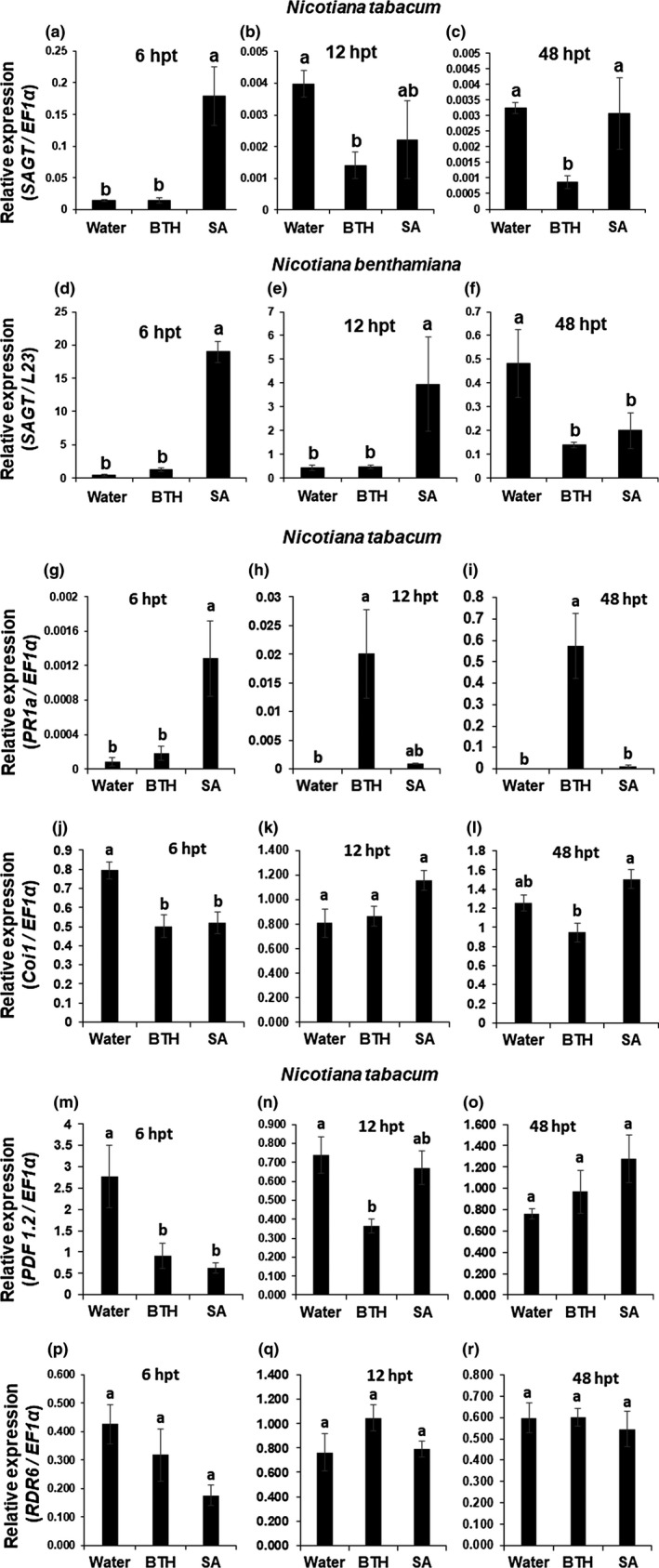
Transcript levels of several resistance‐related genes in benzothiadiazole (BTH)‐treated wild‐type *Nicotiana tabacum* and *N. benthamiana* plants. (a)–(c), (g)–(r) Wild‐type (Wt) *N. tabacum*, (d)–(f) Wt *N. benthamiana* plants. (a)–(f) *SAGT*, (g)–(i) *PR1a*, (j)–(l) *Coi1*, (m)–(o) *PDF1.2*, (p)–(r) *RDR6*. Leaf samples were harvested at 6, 12, and 48 hr post‐treatment (hpt) with BTH (0.12 mM) or salicylic acid (SA) (1 mM). Mean relative transcript levels (±*SE*) of the host genes were measured by quantitative reverse transcription PCR. Levels (in arbitrary units) are for individual plants (*n* = 4). Different letters denote statistically significant differences among the BTH‐, SA‐ and water‐treated leaves (Tukey–Kramer method; *p* < .05). Bars indicate standard errors (±*SE*)

Focusing on the genes with a significant change in transcript levels during the 12 days of observation, we noticed that only *SAGT* transcription was continuously down‐regulated in the BTH‐treated Wt Nt plants in the assay (Figure 2). We thus decided to elucidate the role of *SAGT* in the BTH‐induced immunity. SAGT catalyses the conversion of SA to SAG, which does not stimulate plant defence responses (Lee and Raskin, 1998). The transcript level of *SAGT* in the BTH‐treated Wt Nt plants compared to the level in the water‐treated plants did not differ at 6 hpt (Figure 3a) but was lower at 12 and 48 hpt (Figure 3b,c). On the contrary, *SAGT* in the SA‐treated Wt Nt plants was greatly up‐regulated at 6 hpt (Figure 3a), then down‐regulated to levels equivalent to those in the water‐treated plants at 12 and 48 hpt (Figure 3b,c). We also used Wt Nb in the same experiments to determine whether the *SAGT* expression profile in response to BTH is similar in other *Nicotiana* species. The results showed that *SAGT* expression was very similar between the two species except that the *SAGT* transcript level in the BTH‐treated Wt Nb plants was almost equivalent to that in the water‐treated controls at 12 hpt, indicating that the initial effect of BTH in Nb can last longer than in Nt (Figure 3d–f). In addition to *SAGT*, we measured the transcript levels of *PR1a, Coi1*, *PDF 1.2*, and *RDR6*. The transcript levels of *PR1a* in the BTH‐treated Wt Nt plants were equivalent to those in the water‐treated controls at 6 hpt (Figure 3g) but were significantly higher at 12 and 48 hpt (Figure 3h,i). On the contrary, *PR1a* in the SA‐treated Wt Nt plants was greatly up‐regulated at 6 hpt (Figure 3g), then quickly down‐regulated to the levels equivalent to those in the water‐treated controls after 12 hpt (Figure 3h,i). These results suggest that the resistance induced by BTH lasts longer than that by SA. In the BTH‐ and the SA‐treated Wt Nt plants, *Coi1*, *PDF 1.2*, and *RDR6* had similar expression profiles (Figure 3j–r).

### 2.3 BTH decreases the level of *SAGT* transcripts and SAG

**Figure 4:**
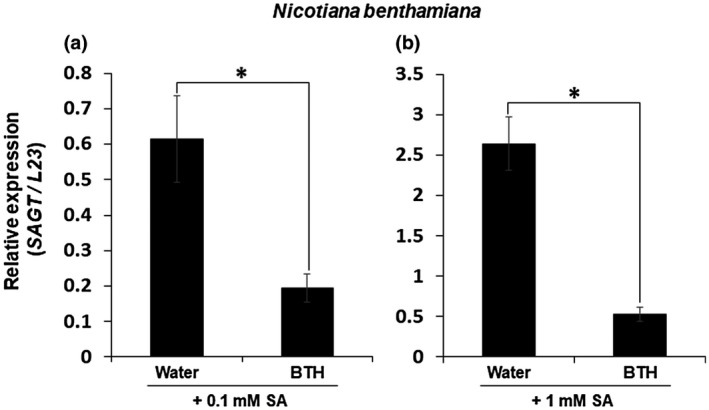
Transcript levels of *SAGT* in wild‐type *Nicotiana benthamiana* plants treated with salicylic acid (SA) after benzothiadiazole (BTH) as determined by quantitative reverse transcription PCR. Leaf tissues were treated with (a) SA (0.1 mM) or (b) SA (1.0 mM) at 42 hr after BTH treatment (0.12 mM), then collected at 6 hr after SA treatment. Levels (in arbitrary units) are for individual plants (*n* = 8). *Significant difference (*p* < .05) between BTH plus SA‐treated and water plus SA‐treated leaves, according to Student's *t* test. Bars indicate standard errors (±*SE*)

We then conducted pre‐BTH + subsequent‐SA applications to investigate whether BTH can reduce the *SAGT* expression level compared to the *SAGT* induction by exogenously applied SA. As shown in Figure 4a,b, even a relatively low level of BTH (0.12 mM) efficiently cancelled the *SAGT* induction by a higher level of SA (1 mM). We therefore assume that BTH can directly and strongly control *SAGT* expression regardless of the concentration of endogenous SA.

**Figure 5:**
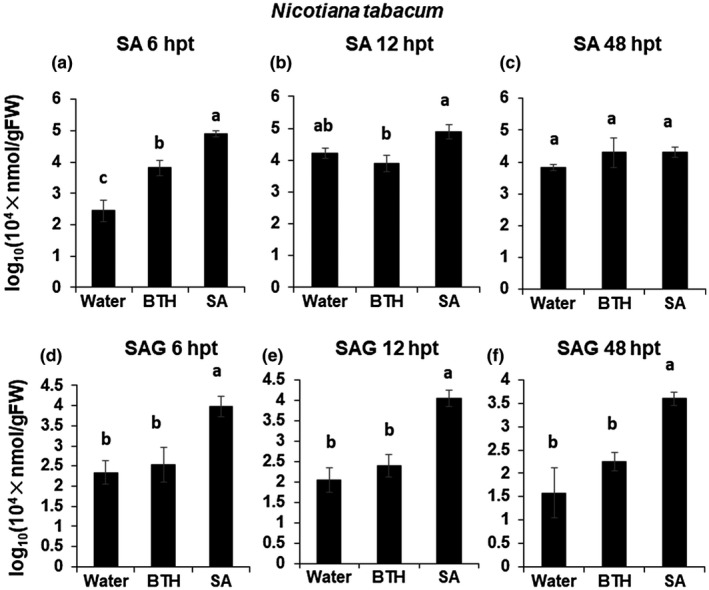
Mean relative levels of salicylic acid (SA) and SA 2‐*O*‐β‐D‐glucoside (SAG) in BTH‐ (or SA‐) treated wild‐type *Nicotiana tabacum* plants. (a)–(f) Wild‐type *N. tabacum* (Wt Nt) plants. Leaf tissues were collected at 6, 12, and 48 hr after treatment with BTH (0.12 mM) or SA (0.1 mM; measurable concentration). Levels (in arbitrary units) are for individual plants (*n* = 4). Different letters denote statistically significant differences among the BTH‐, SA‐, and water‐treated leaves (Tukey–Kramer method; *p* < .05). Bars indicate standard errors (±*SE*)

Next, the levels of SA and SAG were measured in the Wt Nt and Wt Nb plants (Figures 5 and S3). In the Nt plants, both the SA and BTH treatments led to a temporal increase in SA at 6 hpt, but SA levels declined to the same levels as in the water‐treated control by 12 hpt. On the contrary, only the SA‐treated plants had much higher SAG levels than in the water‐treated controls at 6, 12, and 48 hpt. The results in the Nb plants were essentially similar except that higher levels of SA seemed to persist longer than in the Nt plants (Figure S3). These results together suggest that the dynamics of *SAGT* regulation by BTH and SA are intrinsically different.

### 2.4 *SAGT* stimulates biomass production and negatively regulates SA and JA levels

**Figure 6:**
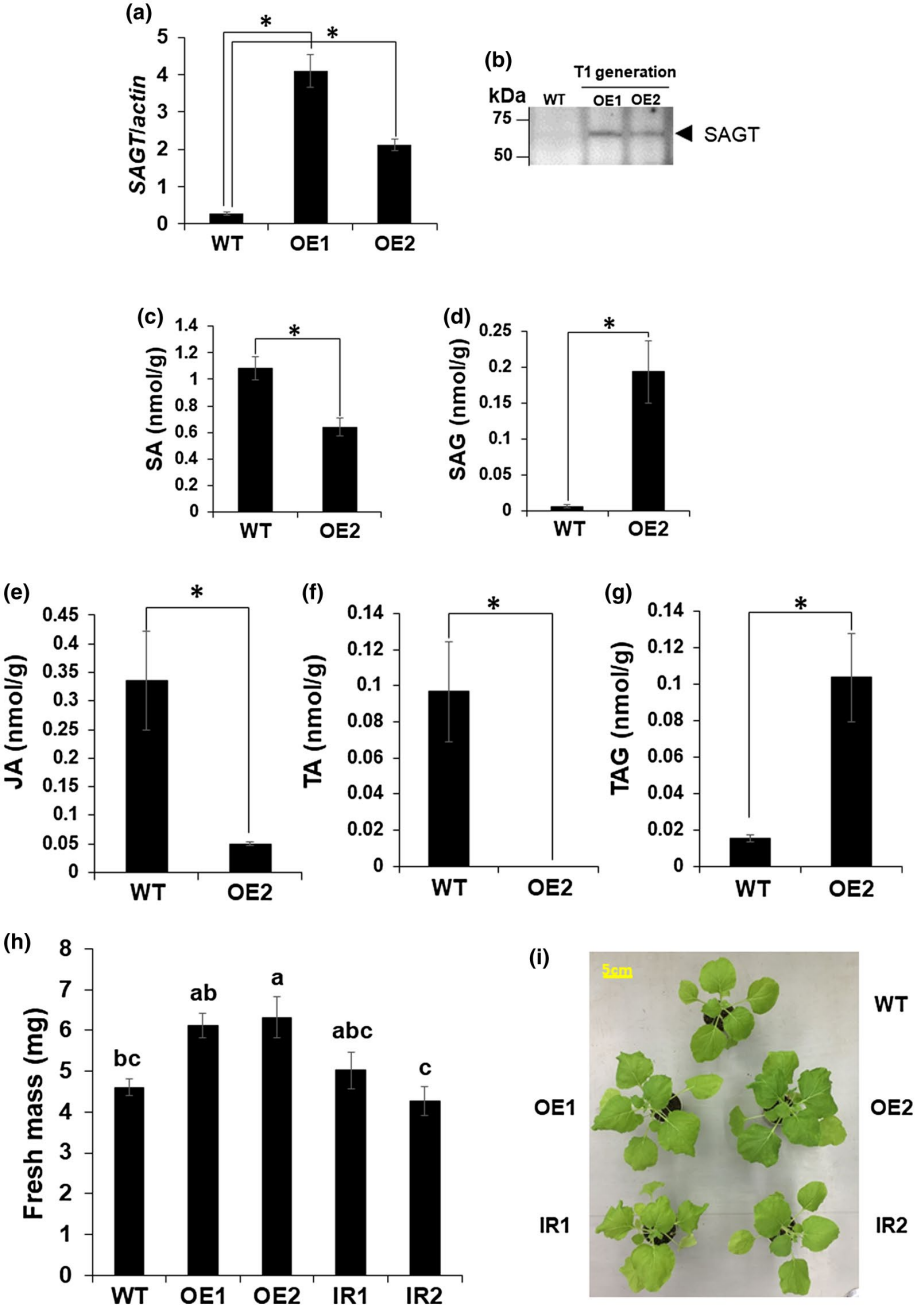
Validation of *SAGT*‐overexpression and *SAGT*‐silenced *Nicotiana benthamiana* (Nb) lines. WT, wild‐type Nb plants. (a) Mean relative transcript levels (±*SE*) of *SAGT* in *SAGT*‐overexpression (OE1 and OE2) Nb lines were measured by quantitative reverse transcription PCR. Levels (in arbitrary units) are for individual plants (*n* = 4). *Significant difference between the OE1 (or OE2) and the WT Nb plants, according to Student's *t* test (*p* < .05). (b) Western blot analysis of SAGT protein overexpressed in OE1‐ and OE2‐transgenic Nb lines. SAGT was detected using anti‐SAGT peptide antibody. (c)–(g) Mean relative levels of (c) salicylic acid (SA), (d) SA 2‐*O*‐β‐D‐glucoside (SAG), (e) jasmonic acid (JA), (f) tuberonic acid (TA), and (g) tuberonic acid glucoside (TAG) (±*SE*) in OE2 Nb plants. Levels (in arbitrary units) are for WT and OE2 plants (*n* = 4). *Significant difference between OE2 Nb line and the WT Nb plants, according to Student's *t* test (*p* < .05). (h) Biomass of wild‐type (WT), *SAGT*‐overexpressing Nb lines (OE1 and OE2), and *SAGT*‐suppressing Nb lines (IR1 and IR2) at 14 days post‐treatment (dpt). Different letters denote statistically significant differences among the WT and the transgenic Nb lines (Tukey–Kramer method; *p* < .05, *n* = 7). (i) WT, OE lines and IR lines at 14 dpt. Whole plants were sprayed with benzothiadiazole (BTH) (0.12 mM)

Because BTH treatment has been previously reported to lead to decreased plant biomass (Canet *et al.*, 2010), we generated *SAGT*‐overexpressing (OE1 and OE2) and *SAGT*‐silenced (IR1 and IR2) Nb transgenic lines to examine whether the *SAGT* accumulation levels, which are suppressed by BTH, alter plant growth. After treatment with BTH, the OE1 and OE2 Nb lines produced higher levels of *SAGT* transcripts and SAGT (Figure 6a,b). As we expected, the transgenic lines also accumulated significantly lower levels of SA and higher levels of SAG than in Wt control plants (Figure 6c,d). Curiously, OE2 plants had less JA and TA and accumulated more tuberonic acid glucoside (TAG) than in the Wt control plants (Figure 6e–g). These observations suggest that JA is converted to TAG via TA by SAGT‐mediated glucosylation, thus reducing JA accumulation (Figure 6e–g), in agreement with Seto *et al. *(2011), who described that SAGT can convert TA into TAG. OE1 plants sprayed with BTH also had slightly more biomass than the Wt control plants, and OE2 plants had significantly more (Figure 6h,i). In contrast, the biomass of IR1 and IR2 plants, which contained lower levels of *SAGT* transcripts (Figure S5), was not significantly greater than that of the Wt control plants after treatment with BTH (Figure 6h,i). These results together indicate that *SAGT* has a key role in the trade‐off between plant defence and biomass production.

### 2.5 A role of *SAGT* in plant immunity

**Figure 7:**
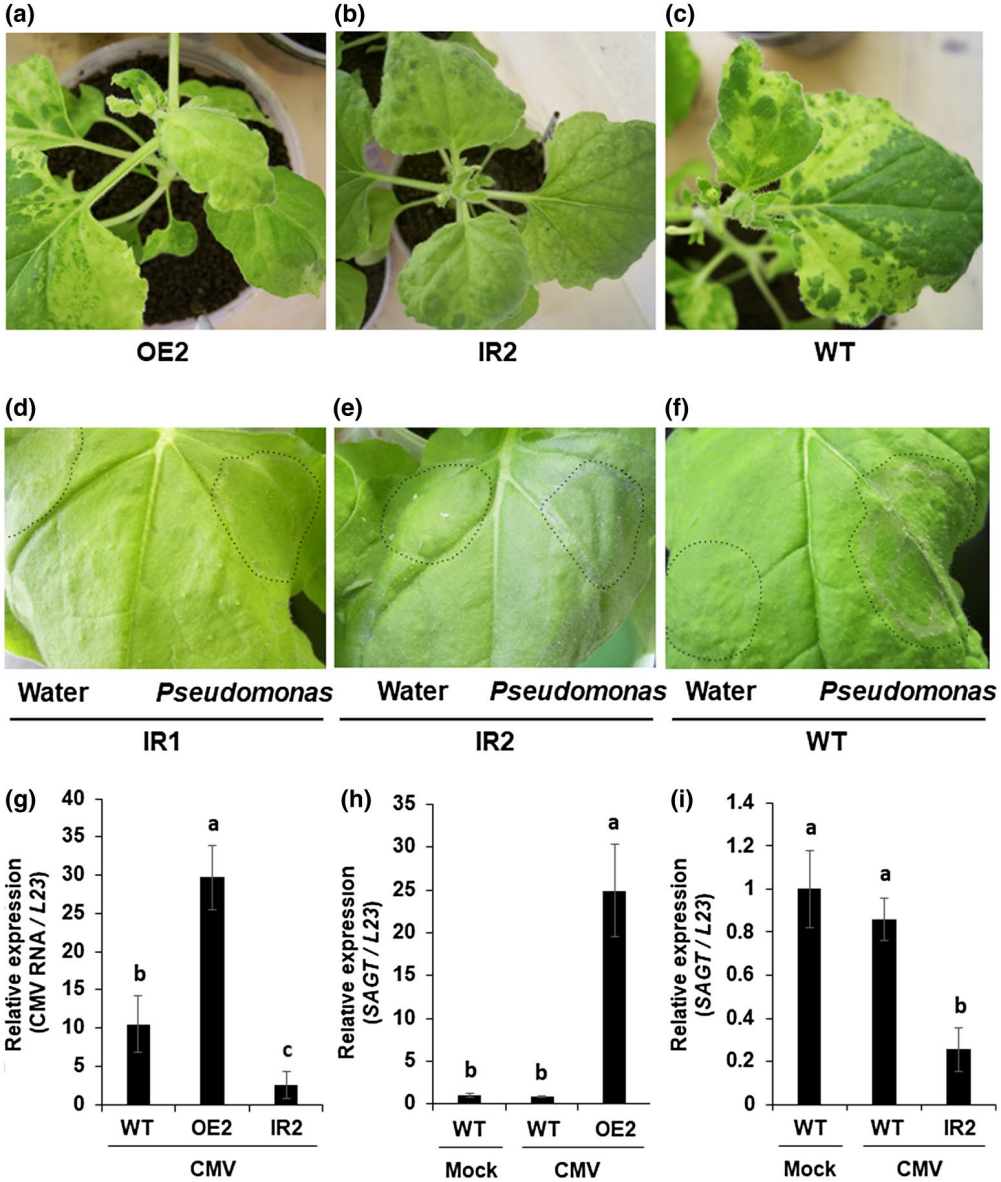
Symptoms, mean relative viral RNA, and transcript levels of *SAGT* in *SAGT*‐overexpressing and the *SAGT*‐silenced *Nicotiana benthamiana* (Nb) lines inoculated with cucumber mosaic virus strain Y (CMV‐Y) and *Pseudomonas syringae*. (a)–(c) Symptoms developed on the Nb transgenic plants (OE2 and IR2 lines) at 21 days post‐inoculation (dpi). (d), (e) Suppression and (f) development of necrosis in the *SAGT*‐suppressing Nb lines (IR1 and IR2) and wild‐type (WT) Nb at 20 hr post‐infiltration of *P. syringae*. Area infiltrated with bacterial suspension or water is indicated by a broken line. (g)–(i) Mean relative levels (±*SE*) of CMV‐Y RNA3 and RNA4, and of *SAGT* transcripts in the upper, noninoculated leaf tissues of the Nb plants at 21 dpi. Relative accumulation levels of viral RNA and *SAGT* transcripts were measured by quantitative reverse transcription PCR using total RNAs from leaves of the Nb plants. CMV RNA levels (in arbitrary units) and *SAGT* transcript levels (in arbitrary units) are for individual plants. Different letters denote statistically significant differences among the healthy WT Nb, CMV‐inoculated WT Nb, and transgenic Nb plants (Tukey–Kramer method; *p* < .05, *n* = 7)

To confirm the direct involvement of *SAGT* in host defensive responses, we inoculated OE2 and IR2 plants with CMV. The OE2 plants developed chlorosis and yellow mosaic more consistently than the control Wt plants did (Figures 7a,c and S6), whereas the symptoms on IR2 plants were somewhat attenuated compared to the severe mosaic on the control at 21 dpi (Figures 7b,c and S6). Similarly, after the inoculation with *Pseudomonas syringae*, the IR1 and IR2 lines expressed a hypersensitive response in the infiltrated areas, indicating that SA‐related host resistance had been activated in the IR plants (Figure 7d–f). CMV accumulation levels were approximately 3‐fold higher in the OE2 plants than those of WT, and one‐quarter the level of WT in the IR2 plants (Figure 7g). We further confirmed that *SAGT* transcript levels were stably up‐regulated in OE2 plants and down‐regulated in IR2 plants at 21 dpi (Figure 7h,i). Other transgenic lines (OE1 and IR1) showed similar results (Figure S7). Taken together, these results demonstrate that *SAGT* has an important role in host defensive responses.

## 3 DISCUSSION

### 3.1 Induction of immunity by BTH does not induce SA synthesis and RNA silencing

In contrast to the strong induction of *PR1a* and *PR1b* gene expression by treatment with BTH, the other five JA/ET‐mediated resistance‐related genes, *Coi1*, *PDF1.2*, *EREBP1*, *EREBP2*, and *ERF1*, were not up‐regulated (Figure [Link], [Link], [Link]). This result is consistent with the reports for *Arabidopsis thaliana* mutants that host resistance‐related genes involved in the JA/ET signal transduction pathways are not key players in BTH‐induced resistance (Lawton *et al.*, 1996). In addition, the results from the comparative quantification of transcript levels of *ICS*, *PAL*, *RDR1*, and *RDR6* suggest that induction of immunity by BTH did not significantly induce biosynthesis of SA and RNA silencing (Figure [Link], [Link], [Link]). Then, what is the key factor for BTH‐induced resistance? What is responsible for the difference between BTH and SA? We thus further analysed the role of *SAGT* in the BTH‐mediated resistance, considering that *SAGT* may be responsible for the difference between BTH and SA (Figure 2).

### 3.2 BTH‐mediated immune induction is enhanced through the suppression of *SAGT* expression

Exogenous application of BTH not only induces resistance against viral infection, but also suppresses symptom development in the Wt Nt and Nb plants (Figure 1). To obtain a clue for the operation site of BTH in the resistance induction, we next analysed comparative changes in expression of the several defence‐related genes in comparison with the case of SA. Among the examined host genes, *PR1a* showed a longer‐lasting induction in the BTH‐treated Wt Nt plants. The result was consistent with prominent up‐regulation of *PR1a* in *A. thaliana* on the basis of microarray analyses (Gruner *et al.*, 2013). We then noticed that BTH, but not SA, specifically suppressed *SAGT* transcription during plant immune induction (Figure 3), providing a feasible explanation for the phenomenon that BTH is more efficient than SA in induction of the SA‐dependent host immune system. The inhibition of the conversion of SA into SAG could lead to high levels of SA accumulation under biotic and abiotic stresses. The *SAGT* transcript levels in *NahG* Nt plants treated with BTH were equivalent to those in the water‐treated *NahG* Nt plants, implying that initial accumulation of SA may be necessary for the down‐regulation of *SAGT* by BTH (Figure [Link], [Link], [Link]). As shown in Figure 5, SA seemed to be induced to some extent at 6 hpt by BTH, although BTH did not seem to induce SAG accumulation in either Wt Nt or Wt Nb plants (Figures 5 and S3). In support of our results, Friedrich *et al. *(1996) previously reported that their BTH treatment elicited host resistance pathways downstream of SA accumulation, not by directly enhancing SA accumulation. We here conclude that one of the crucial roles of BTH is the suppression of *SAGT* transcript levels to inhibit the conversion of SA to SAG. The observation that BTH is a stronger inducer of resistance than SA can be at least partially explained by the finding that BTH has a slightly higher affinity than SA for NPR1 (Wu *et al.*, 2012); NPR1 is central to the activation of the SA‐mediated signalling pathway. Our hypothesis based on *SAGT* provides an additional explanation for the BTH‐mediated immune induction. We thus believe that BTH can function not only as a simple SA mimic, but also as an efficient suppressor of *SAGT* transcription.

### 3.3 *SAGT* contributes to the trade‐off between plant growth and defence

The effect of SA on plant growth varies among plant species. Exogenous application of SA promotes growth of soybean plants (Gutiérrez‐Coronado *et al.*, 1998) and larger ears of wheat (Shakirova *et al.*, 2003). A low level (50 µM) of SA has a positive effect on growth of chamomile plants, but a high level (250 µM) has negative effect (Kovácik *et al.*, 2009). In addition, the exogenous SA application (100 µM and 1 mM) suppressed the development of trichomes in *A. thaliana* (Traw and Bergelcon, 2003). Exogenous SA also causes a change in hormonal balance to affect photosynthesis, transpiration, and opening and closure of stomata (Shakirova *et al.*, 2003; Stevens *et al.*, 2006; Abreu and Munné‐Bosch, 2009). An appropriate level of SA may be indispensable to properly regulate plant growth rate; SA is even involved in flowering and senescence (Khurana and Cleland, 1992; Morris *et al.*, 2000). We here hypothesize that *SAGT* is a key player in the regulation of SA level and thus in plant growth.

The *SAGT*‐overexpressing Nb lines, OE1 and OE2, had increased SAG levels and plant fresh mass, whereas the *SAGT*‐silenced Nb lines, IR1 and IR2, did not (Figure 6). These results suggest that *SAGT* has a crucial role in optimizing plant fitness by allocating resources for resistance activation and biomass production by regulating SA and SAG levels. In a study of the effects of the SA‐mediated signal transduction pathway on biomass production using BTH, Canet *et al. *(2010) reported that plant fresh mass of *A. thaliana* was reduced after BTH treatment, which they concluded to be due to the stimulation of the SA‐mediated signal transduction pathway, not to BTH phytotoxicity. In addition, SA and BTH also inhibited the auxin‐mediated signal transduction pathway, and *AXR3*, the auxin‐inducible AUX/IAA transcription regulator, played a key role as the sensor for SA and BTH in controlling the balance between disease resistance and plant growth (Canet *et al.*, 2010).

Canet *et al. *(2010) further described that the biomass of BTH‐treated *A. thaliana* plants was reduced in a dose‐dependent manner. Acibenzolar, a converted form of BTH, itself thus seems to be maintained at a constant level without conversion to SAG in cells. Although endogenous SA in the BTH‐treated plants was lower than in the SA‐treated plants, the level had increased to some extent by 6 hpt (Figure 5). Because BTH suppresses transcription of *SAGT* in the BTH‐treated plants (Figures 3 and 4), leading to an increase in endogenous SA, an additive effect of the elevated endogenous SA and exogenously supplied BTH may cause growth inhibition.

In contrast to an increase in SAG levels, the SA levels in the OE Nb lines were reduced by half by overexpression of *SAGT*. BTH treatment of *SAGT*‐overexpressing Nb plants led to a slight increase in biomass compared with the Wt Nb plants (Figure 6). In the transgenic Nb plants, the inhibitory effect of BTH on *SAGT* expression must have been hampered by the overexpression of *SAGT*. On the contrary, the biomass of the Wt Nb plants will be reduced by the additive effect of BTH and endogenous SA. Considering the results together, we attribute the difference in biomass to the growth reduction in the Wt Nb plants as a result of low levels of SAGT, not to growth enhancement by high levels of SAGT in the *SAGT*‐overexpressing Nb plants.

**Figure 8:**
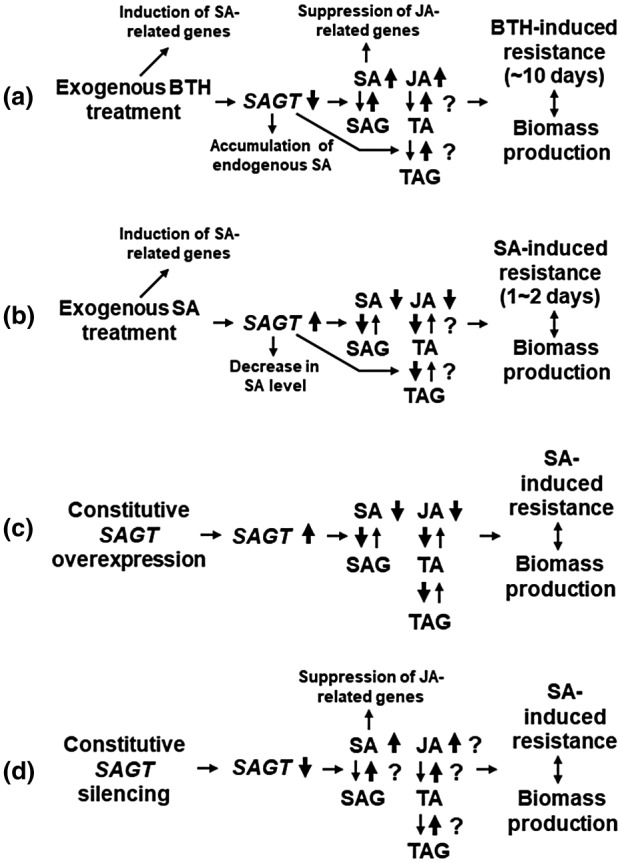
Model for important roles of *SAGT* in plant responses to benzothiadiazole (BTH) and salicylic acid (SA). Bold vertical arrows indicate the direction of regulation. (a) Exogenous BTH treatment for plants. (b) Exogenous SA treatment for plants. (c) Constitutive *SAGT* expression in transgenic plants. (d) Constitutive *SAGT* silencing in transgenic plants

We found that BTH can down‐regulate *SAGT* expression and thus reduce SAG accumulation, unlike SA, as illustrated in our hypothetical model in Figure 8a,b. In this model, we postulate that BTH can up‐regulate endogenous levels of both SA and JA by suppressing SAGT synthesis, although a high level of endogenous SA eventually inhibits JA‐mediated gene expression (Caarls *et al.*, 2015) for approximately 10 days as estimated from Figure S1a,b, resulting in induction of the SA‐related defence responses and subsequently lower biomass (Figure 8a). On the contrary, exogenously applied SA can up‐regulate the expression level of *SAGT*, which converts SA to SAG, and also TA to TAG, leading to the inhibition of both SA‐ and JA‐mediated resistance (Figure 8b) and eventually transient defence responses for approximately a couple of days as estimated from Figure 3g–i. Considering that *SAGT* is the key factor in the control of SA and JA levels, the use of BTH for studies on plant defence responses will be an excellent tool to understand the trade‐off between plant defence and biomass production for optimum fitness.

Using the *SAGT*‐overexpressing and ‐silencing Nb lines, we demonstrated that *SAGT* regulates the balance between plant defence and growth (Figure 8c,d). In this model, the constitutive overexpression of *SAGT* can simultaneously down‐regulate both SA‐ and JA‐mediated resistances, resulting in greater biomass production (Figure 8c). On the contrary, the constitutive silencing of *SAGT* can up‐regulate SA‐mediated resistance but reduce biomass production (Figure 8d). Although the involvement of JA in biomass production has not been intensively discussed before, unlike SA, there are still some findings in connection with this. For example, Campos *et al. *(2016) previously reported that attenuated growth of *A. thaliana* associated with anti‐insect resistance could be caused by the JA‐mediated signalling. Given the high relevance of *SAGT* to the SA/JA‐mediated defence, it is possible that *SAGT* has roles not only in pathogen‐induced but also in herbivory‐induced resistance, both of which are perhaps associated with reduced plant growth. However, further information on the relationship between *SAGT* and JA would be necessary to clearly understand how SA (BTH)/JA controls the balance between plant growth and defence.

In the pathogen inoculation experiments, we found that the CMV accumulation levels were higher in the *SAGT*‐overexpressing plants and lower in the *SAGT*‐silencing plants, which can be explained in our model (Figure 7g–i). In addition, after *Pseudomonas* inoculation, expanding necrotic lesions developed on the inoculated leaves of Wt Nb plants, while necrotic lesions were not obvious on the *SAGT*‐silenced Nb plants at 12 hpi (Figure 7d–f). Numerous papers support our observations. For example, Song *et al. *(2008) showed that transgenic *A. thaliana* plants overexpressing *AtSGT1* were more susceptible to *P. syringae* than the wild type. Noutoshi *et al. *(2012) reported that inhibitors of SAGT actually enhanced resistance against *P. syringae*. Yao *et al. *(2007) also demonstrated that transgenic tobacco overexpressing the β‐glucosidase gene from *Butyrivibrio fibrisolvens* H17c increased SA accumulation through the conversion of SAG into SA, leading to enhancement of host resistance to TMV.

On the contrary, this model does not seem to fit the case of rice. Umemura *et al. *(2009) previously documented that RNAi suppression of *OsSGT1*, a probenazole‐responsive UDP‐glucose:SA glucosyltransferase of rice, impaired probenazole‐dependent disease resistance against blast disease in rice plants, indicating that the role of the ratio of SA/SAG in rice seems to be different from that in other plants. In fact, SAG in rice can induce resistance as SA does (Bundó and Coca, 2016). In rice, SA‐mediated resistance is activated not only by SA but also by SAG, and SAG is differentially induced, suggesting that SAG is a key factor for SA‐mediated rice resistance rather than SA per se (Bundó and Coca, 2016). We therefore presume that the role of SAG in rice is unique compared to other plants.

Gruner *et al. *(2013) previously reported that biologically induced SAR and the immunity induced by exogenously applied BTH shared a common signal transduction system for immune response but with some differences. According to their analyses, some UDP‐glucosyltransferase (SAGT synonym) genes of *A. thaliana* were up‐regulated and others were down‐regulated by either BTH or SA treatment; the individual genes were thus differentially expressed in response to BTH and SA. We here assume that a certain SAGT among the isoforms actively and specifically responds to pathogen infection; the induction of each *SAGT* gene would therefore differ depending on the pathogen. For example, Lee and Raskin (1999) demonstrated that tobacco *SAGT* was specifically induced after inoculation with avirulent viral and bacterial pathogens. Taken together, we conclude that the *SAGT* genes at least in two *Nicotiana* species play an important role in the trade‐off between plant defence and growth.

## 4 EXPERIMENTAL PROCEDURES

### 4.1 Plants, pathogens, and chemical treatment

*N. tabacum* and *N. benthamiana* plants were grown and maintained in an air‐conditioned greenhouse at 25/20 °C (day/night) (Figures 2, [Link], [Link], [Link], and [Link], [Link], [Link]) or in a growth chamber at 25 °C with a 12‐hr photoperiod. The *NahG*‐expressing transgenic *N. tabacum* line was generated essentially using the protocol of Bi *et al. *(1995), and the *N. benthamiana* line was described by Asai *et al. *(2010). Wild‐type CMV‐Y (Suzuki *et al.*, 1991) was propagated and purified as described by Takeshita *et al. *(2012). The largest leaf on a plant of *N. benthamiana* was infiltrated with a bacterial suspension of *P. syringae* according to Krzymowska *et al. *(2007)*,* and kept at 25 °C.

The largest leaf on plants of *N. tabacum* and *N benthamiana* was dipped into BTH (Syngenta Japan K. K., Tokyo, Japan) or SA (1.0 or 0.1 mM) for 5 s without a water rinse after treatment. In the biomass assay, the whole plants of the transgenic *N. benthamiana* lines were sprayed with BTH (Figure 6). BTH was used at 25 ppm (0.12 mM) because *PR1* transcript accumulation in tobacco sprayed with 0.036 mM BTH can peak at levels equivalent to those with 1.2 mM BTH even at 7 days after treatment (Friedrich *et al.*, 1996), and some of the tobacco leaves treated with BTH at 100 ppm (0.476 mM) hardened slightly in our preliminary experiments. SA, whose methylated derivative (MeSA) is volatile and is excreted from plants (Kumar, 2014), was also used at a higher concentration (1 mM) in some experiments (Figures 3 and 4) as done by Lewzey and Carr (2009), Naylor *et al. *(1998), and Zhou *et al. *(2014).

### 4.2 RNA extraction and RT‐qPCR analysis

Total RNA was extracted from leaf tissue with RNAiso Plus (Takara Bio) according to the user manual. Six biological replicates per experimental plot were used to validate gene expression by comparative reverse transcription‐quantitative PCR (RT‐qPCR). First‐strand cDNA was synthesized using 50–200 ng of total RNA and ReverTra Ace qPCR RT Master Mix with gDNA Remover (Toyobo), which contains oligo(dT) primer and random hexamer primer. A *SAGT*‐specific primer was used for efficient reverse transcription of *SAGT*. Comparative qPCR targeting of a host gene and CMV RNA was performed by using THUNDERBIRD SYBR qPCR Mix (Toyobo) and the Thermal Cycler Dice RealTime System Single TP850 (Takara Bio) according to the procedure of the manufacturer and Takeshita *et al. *(2013). Each reaction mixture (total 20 μl) was prepared using 10 μl of THUNDERBIRD SYBR qPCR Mix, 1 μl of 10 pmol of each forward and reverse primer, 1 μl of cDNA template, and 7 μl of water. Primers used in RT‐qPCR are listed in Table S1. Samples were analysed by relative RT‐qPCR as done by Takeshita *et al. *(2013). Transcript accumulation of each target gene was normalized by the quantification of *EF1*α from *N. tabacum* and *L23* from *N. benthamiana*. The *EF1*α and *L23* were selected as normalizers after analyses by geNorm essentially according to Takeshita *et al. *(2013). These values were calculated according to the ΔΔ*C*_t_ method (Pfaffl *et al.*, 2002) and plotted with standard errors. The data were obtained from each assay repeated independently (*n* = 3–8).

### 4.3 Measurement of phytohormones

Leaf tissues were weighed immediately after harvest, then frozen in liquid nitrogen and stored at –80 °C until measurement of SA and SAG using ultraperformance liquid chromatography‐tandem mass spectrometry (UPLC‐MSMS). Leaf samples were prepared and analysed with UPLC‐MSMS as done by Matsuura *et al. *(2009) and Fujiwara *et al. *(2016).

### 4.4 Construction of *SAGT*‐overexpressing and *SAGT*‐silenced transgenic plants

For the *SAGT*‐overexpressing transgenic Nb lines, the PCR‐amplified *SAGT* ORF (1,436 bp) sequence was amplified using primer pair SAGT‐F and SAGT‐R (Table S1). The fragment was cloned into pENTR/D‐TOPO (Thermo Fisher Scientific). The entry clone was integrated into the binary vector pBI‐OX‐GW (Inplanta Innovations, Inc.), using the GATEWAY system (Thermo Fisher Scientific). The recombinant pBI‐SAGT binary plasmid was transferred into *Agrobacterium tumefaciens* LBA4404. Nb plants were transformed by the conventional leaf disk transformation method and selected on kanamycin‐containing Murashige‐Skoog medium as done by Fukuzawa *et al.* (2018). Two *SAGT*‐overexpressing lines (OE1 and OE2) were selected from 38 T_1_ lines regenerated from T_0_ generation. The T_1_ lines of OE1 and OE2 were confirmed to be homozygous.

For the *SAGT*‐silenced transgenic Nb lines, plasmid pBI‐IR‐SAGT (Figure S4) containing an inverted repeat of the 600‐nt *SAGT* sequence was constructed. The PCR‐amplified 600‐nt *SAGT* sequence was amplified using primer pair SAGT‐210F and SAGT‐809R (Table S1). The fragment was then cloned into pENTR/D‐TOPO (Invitrogen). The entry clone was integrated into the binary vector, pBI‐sense, antisense‐GW (Inplanta Innovations Inc.) using the GATEWAY system. The recombinant pBI‐IR‐SAGT vector was transferred into *A. tumefaciens* LBA4404. Nb plants were transformed as described above. Two independent *SAGT*‐silenced lines (IR1 and IR2) were selected from 113 regenerated lines. Reduced transcript levels of *SAGT* in the two lines at the T_0_ generation stage were verified by RT‐qPCR (Figure S5). Seeds from the two selected T_0_ transgenic lines were collected, and homozygous T_1_ lines were used for the subsequent experiments.

### 4.5 Nucleotide sequencing

The nucleotide sequences of the RT‐qPCR product and transgene were verified using the Big Dye Terminator DNA Sequencing Kit v. 3.1 (Applied Biosystems) and the ABI Prism 310 Genetic Analyzer. The sequence was analysed using the program GENETYX‐Win v. 10 (GENETYX Corp.).

## Supporting information


**FIGURE S1** Mean relative transcript levels of resistance‐related genes in BTH‐treated and water‐treated *Nicotiana tabacum* plants

**FIGURE S2** Mean relative transcript levels of resistance‐related genes in BTH‐treated and water‐treated *NahG*‐transgenic* Nicotiana tabacum* plants (NahG Nt)

**FIGURE S3** Mean relative levels of SA and SAG in BTH‐ (or SA‐) treated wild‐type *Nicotiana benthamiana* plants

**FIGURE S4** Schematic representation of pBI‐IR‐SAGT plasmid used to construct *SAGT*‐silenced transgenic *Nicotiana tabacum* lines

**FIGURE S5** Validation of *SAGT*‐silenced *Nicotiana benthamiana* lines

**FIGURE S6** CMV‐Y‐induced symptoms on *SAGT*‐overexpressing (OE2) and ‐silenced (IR2) *Nicotiana benthamiana* lines

**FIGURE S7** Symptoms, mean relative viral RNA and transcript levels of *SAGT* in *SAGT*‐overexpressing (OE1) and the *SAGT*‐silenced (IR1) *Nicotiana benthamiana* lines infected with CMV‐Y

**TABLE S1** Oligonucleotide primers used in RT‐qPCR analyses and construction of transgenic plants

## Data Availability

The data that support the findings of this study are available from the corresponding author upon reasonable request.
