# Supplementary figures and images for: Role of salicylic acid glucosyltransferase in balancing growth and defence for optimum plant fitness
Source: Mol Plant Pathol. 2020 Jan 21;21(3):429–42. doi: 10.1111/mpp.12906 (PMC7036366; doi:10.1111/mpp.12906)

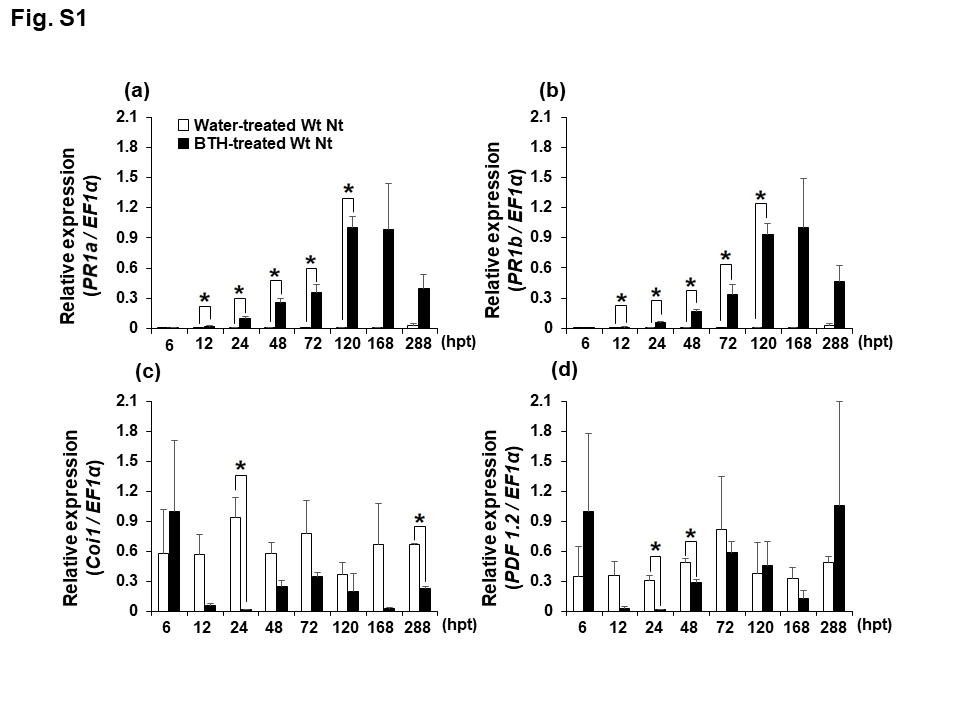

Supplement: Supplementary file 1 — FIGURE S1 Mean relative transcript levels of resistance‐related genes in BTH‐treated and water‐treated Nicotiana tabacum plants [file MPP-21-429-s001.tif]

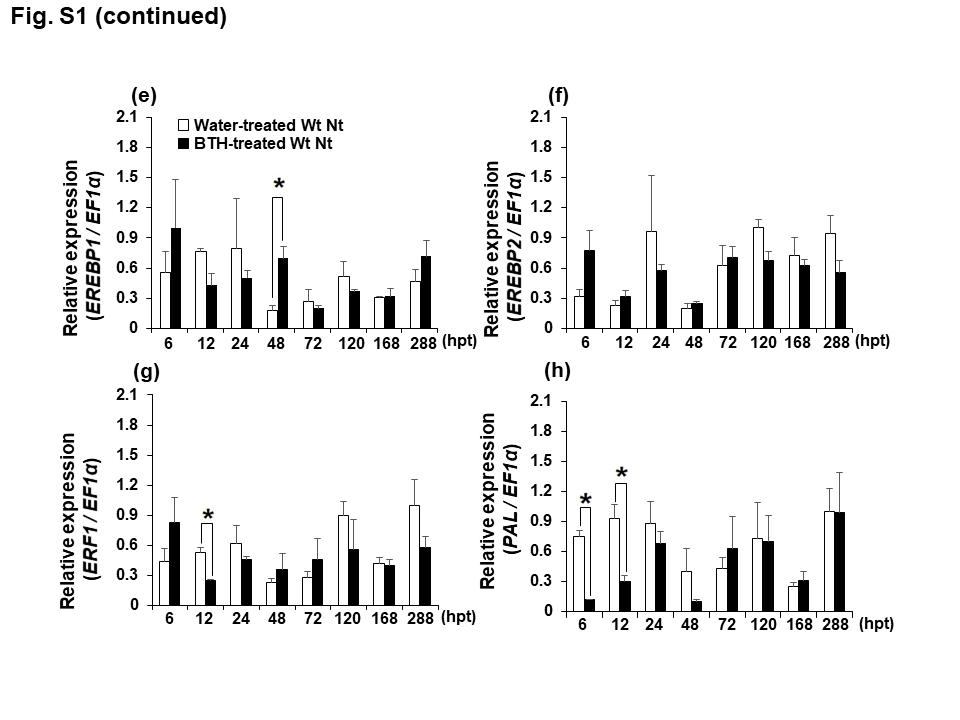

Supplement: Supplementary file 2 [file MPP-21-429-s002.tif]

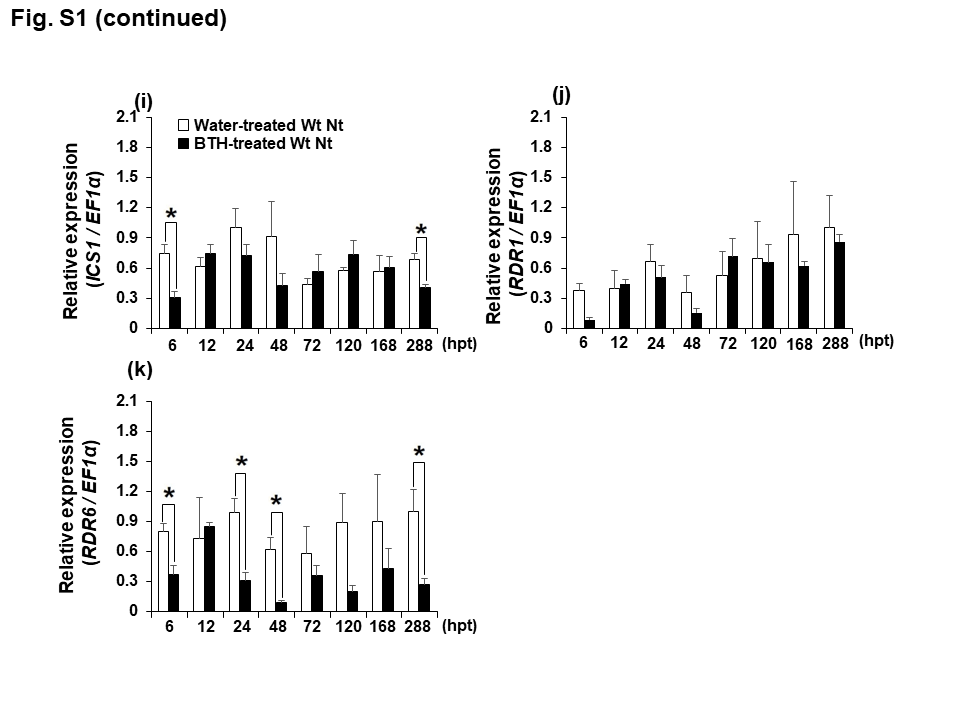

Supplement: Supplementary file 3 [file MPP-21-429-s003.tif]

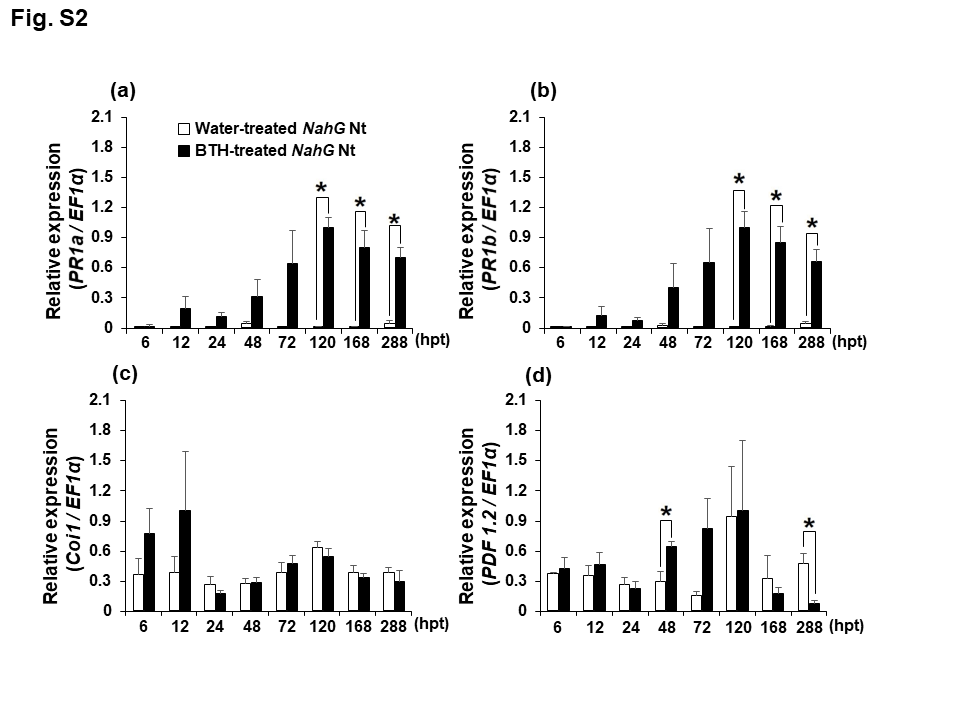

Supplement: Supplementary file 4 — FIGURE S2 Mean relative transcript levels of resistance‐related genes in BTH‐treated and water‐treated NahG‐transgenic Nicotiana tabacum plants (NahG Nt) [file MPP-21-429-s004.tif]

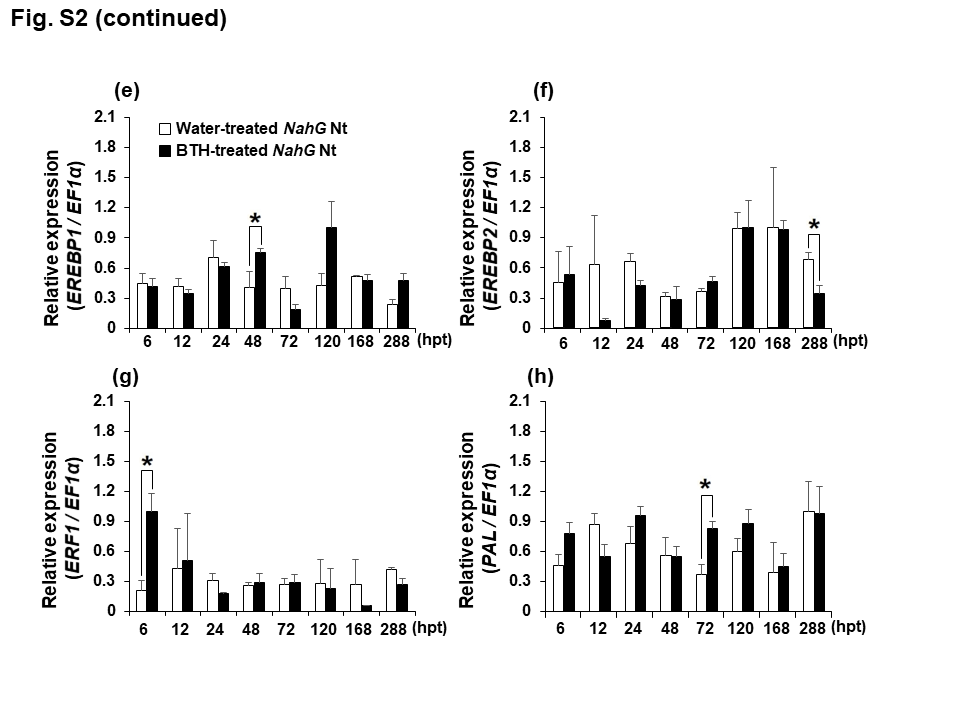

Supplement: Supplementary file 5 [file MPP-21-429-s005.tif]

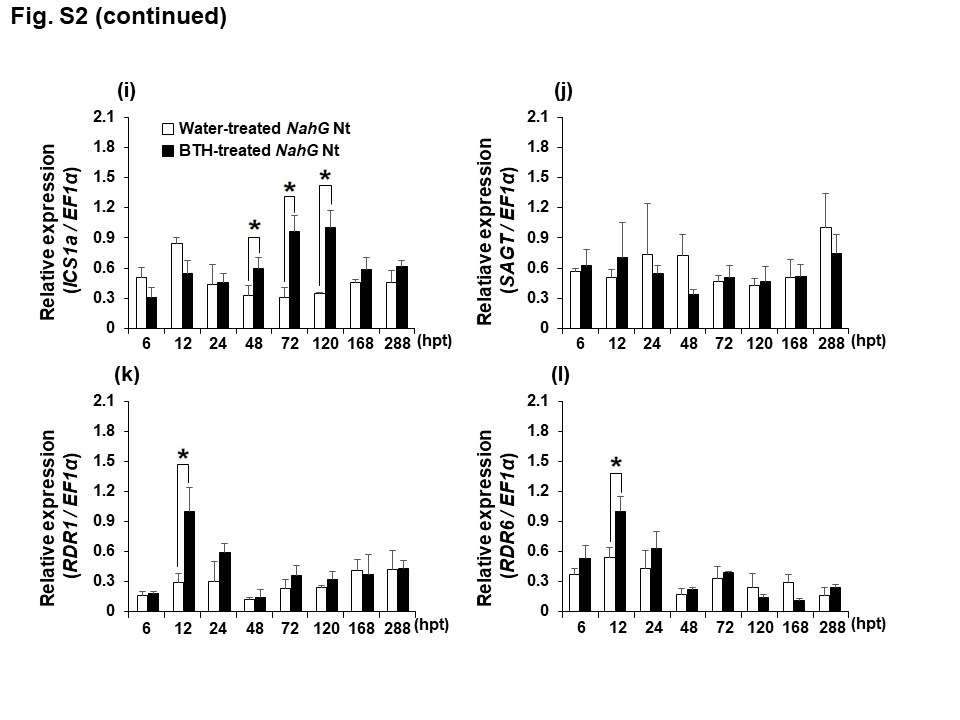

Supplement: Supplementary file 6 [file MPP-21-429-s006.tif]

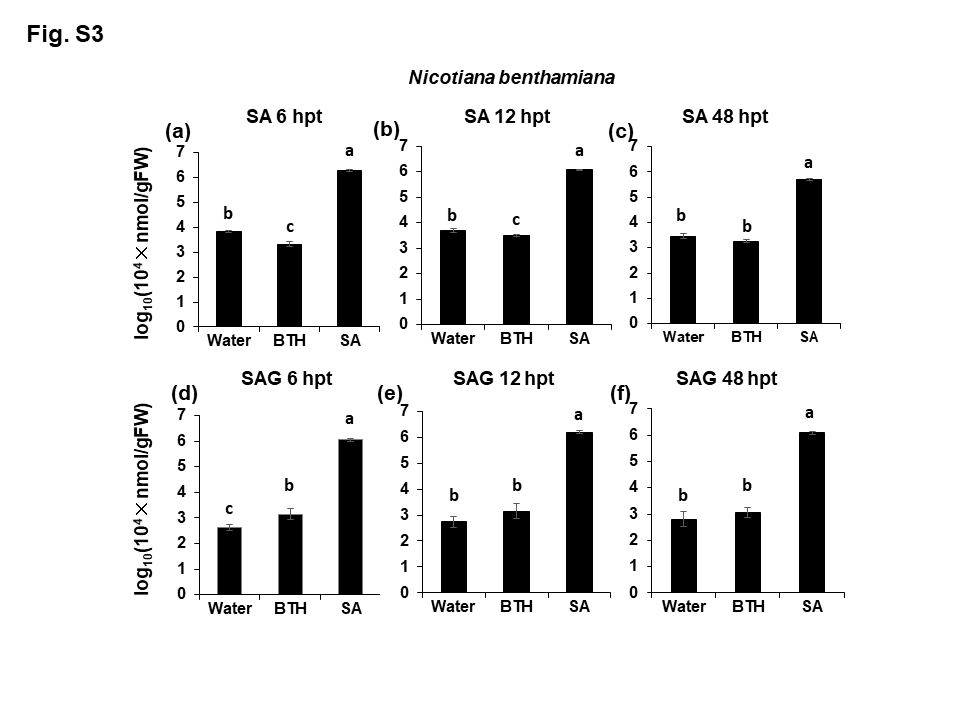

Supplement: Supplementary file 7 — FIGURE S3 Mean relative levels of SA and SAG in BTH‐ (or SA‐) treated wild‐type Nicotiana benthamiana plants [file MPP-21-429-s007.tif]

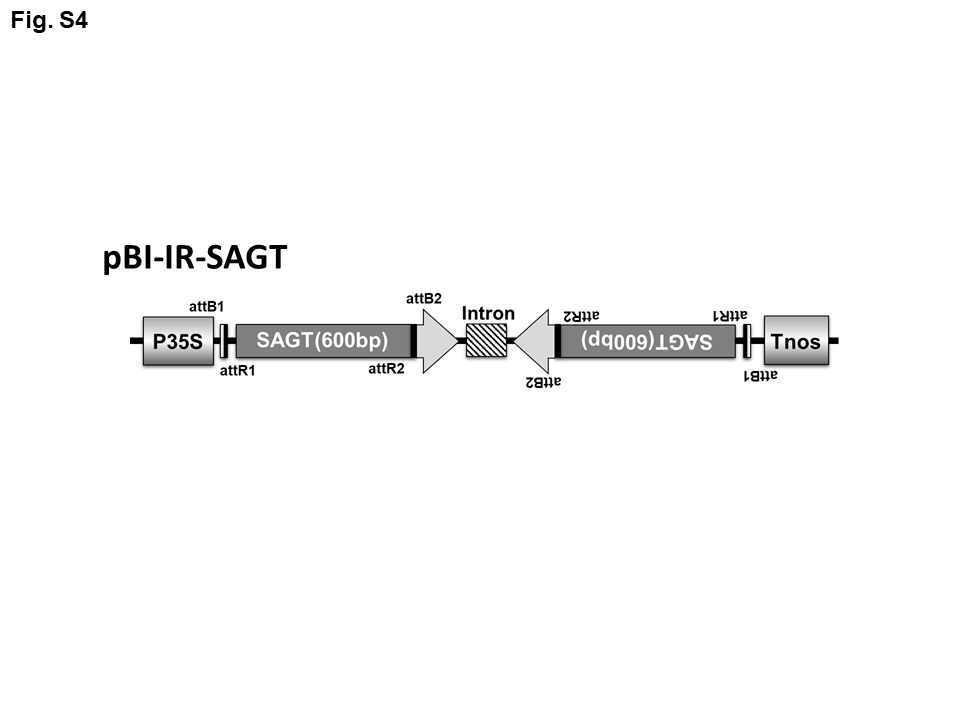

Supplement: Supplementary file 8 — FIGURE S4 Schematic representation of pBI‐IR‐SAGT plasmid used to construct SAGT‐silenced transgenic Nicotiana tabacum lines [file MPP-21-429-s008.tif]

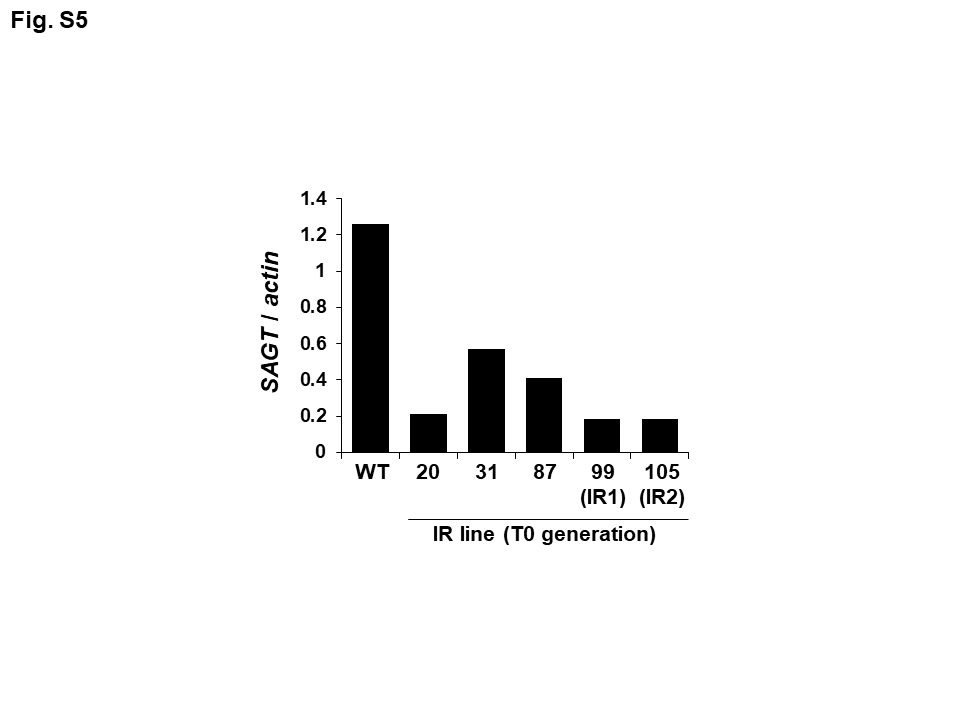

Supplement: Supplementary file 9 — FIGURE S5 Validation of SAGT‐silenced Nicotiana benthamiana lines [file MPP-21-429-s009.tif]

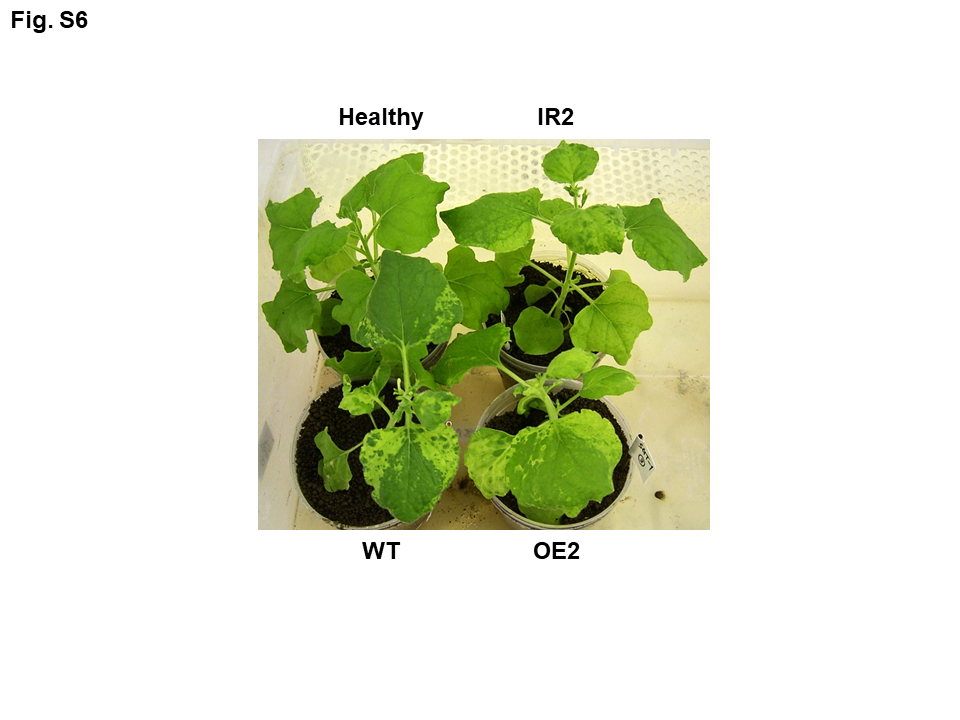

Supplement: Supplementary file 10 — FIGURE S6 CMV‐Y‐induced symptoms on SAGT‐overexpressing (OE2) and ‐silenced (IR2) Nicotiana benthamiana lines [file MPP-21-429-s010.tif]

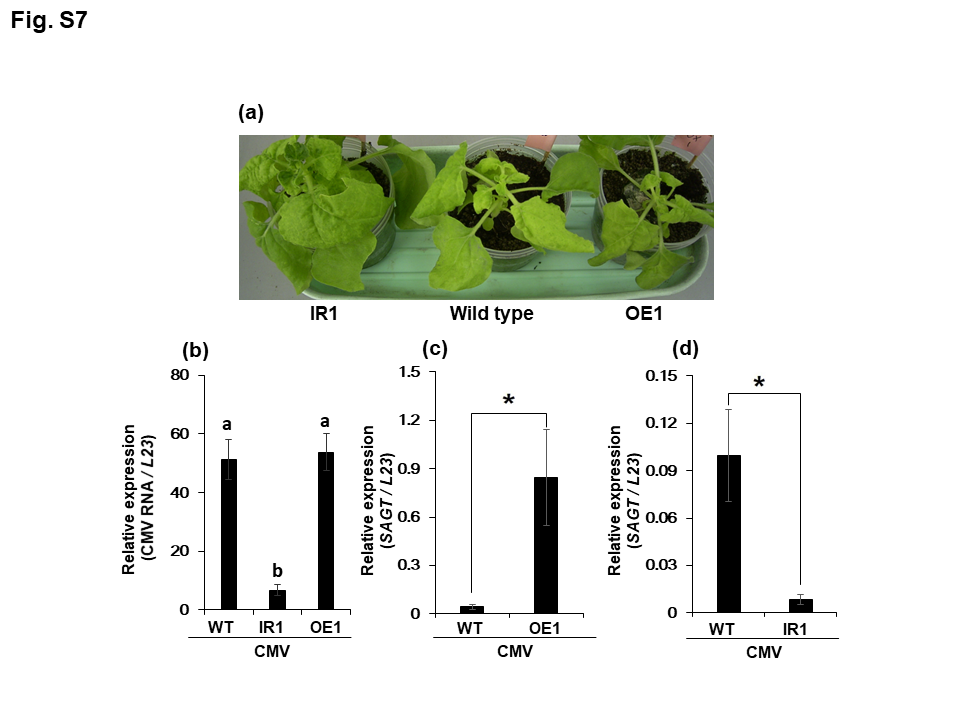

Supplement: Supplementary file 11 — FIGURE S7 Symptoms, mean relative viral RNA and transcript levels of SAGT in SAGT‐overexpressing (OE1) and the SAGT‐silenced (IR1) Nicotiana benthamiana lines infected with CMV‐Y [file MPP-21-429-s011.tif]
