# Supplementary material for: Role of salicylic acid glucosyltransferase in balancing growth and defence for optimum plant fitness
Source: Mol Plant Pathol. 2020 Jan 21;21(3):429–42. doi: 10.1111/mpp.12906 (PMC7036366; doi:10.1111/mpp.12906)
Supplement: Supplementary file 12 — TABLE S1 Oligonucleotide primers used in RT‐qPCR analyses and construction of transgenic plants [file MPP-21-429-s012.pdf]

Table S1 Oligonucleotide primers used in RT-qPCR analyses

Primer name	Sequence (5' - 3')	Accession number
NtPR1a F	ATGCGCAAAATTATGCTTCC	X06930
NtPR1a R	CATTGACCCACATCTCAACG	
NtPR1b F	GATGTAGGCGTGGAACCATT	X03465
NtPR1b R	TTGGCCATGAGAATGTACGA	
NtCoi1 F	GGTGCAGTTACGCACAGAGG	AB433899
NtCoi1 R	CCTCTCGGTCAAGCAAAACC	
NtPDF 1.2 F	CTACCGAGATGGGACCAATG	AB034956
NtPDF 1.2 R	ATCCTTCGGTCAGACAAACG	
NtEREBP1 F	TTGCAGCTGAGAAATCTGGA	AF057373
NtEREBP1 R	AACTTCGGAGCGCAAGATTA	
NtEREBP2 F	GTCTCATGCCATGTTTGACG	D38126
NtEREBP2 R	AGGAATCGTAGCTGGCTCAA	
NtERF1 F	GCGGTTCAAAGGCTCATTTA	D38123
NtERF1 R	TGCAACAGCCTTTCTTCTCC	
NtPAL F	CACCAATTGGTGTCTCTATGG	AB289452
NtPAL R	GTCAGATTAGATGGCAACCC	

Table S1 Oligonucleotide primers used in RT-qPCR analyses (continued)

Primer name	Sequence (5' - 3')	Accession number
NtICS F	CTCCAGCAGTTTGTGGGTATC	AY740529
NtICS R	TCCAAACCAACCAACAGGAC	
NtSAGT F	GTCGTGTCATGGTGTCCACAATTAC	AF190634
NtSAGT R	CTGGTCTGACCAATGTGGCATTG	
NtRDR1 F	GCATTGAACACGCCTTGGA	AJ011576
NtRDR1 R	GCAGAACCCGATTGGATACG	
NtRDR6 F	CTCAGCTTGGGGACCTCA	FJ966891
NtRDR6 R	CAGCCTCCAGAATCCTCAC	
NtEF1α F	CCACACCTCCCACATTGCTGTCA	D63396
NtEF1α R	CGCATGTCCCTCACAGCAAAAC	
NbSAGT F [a)]	GCAATTTACAGGACAACCACG	AF190634
NbSAGT R [b)]	TATGATGATGGTGGCCGTGAG	
NbSAGT 210F	CACCGCAAGCTGGAACCTTTGTGG	
NbSAGT 809R	ACTGAGCTAGCTGGTTGATGG	
NbL23 F [c)]	AAGGATGCCGTGAAGAAGATGT	XM_016619600
NbL23 R [d)]	GCATCGTAGTCAGGAGTCAACC	

a) to d): The specificity of the nucleotide sequence was confirmed by BLAST analyses based on AF190634 and XM_016619600 at the website of "Nicotiana benthamiana Genome & Transcriptome" (benthgenome.qut.edu.au) and by nucleotide sequencing of the qPCR products.
